# Implementing culturally competent transplant care and implications for reducing health disparities: A prospective qualitative study

**DOI:** 10.1111/hex.13124

**Published:** 2020-10-09

**Authors:** Elisa J. Gordon, Elida Romo, Daniela Amórtegui, Alejandra Rodas, Naomi Anderson, Jefferson Uriarte, Gwen McNatt, Juan Carlos Caicedo, Daniela P. Ladner, Michelle Shumate

**Affiliations:** ^1^ Division of Transplantation, Department of Surgery Northwestern Medicine Chicago IL USA; ^2^ Northwestern University Transplant Outcomes Research Collaborative (NUTORC), Comprehensive Transplant Center, Feinberg School of Medicine Chicago IL USA; ^3^ Center for Health Services and Outcomes Research Northwestern University Feinberg School of Medicine Chicago IL USA; ^4^ Center for Bioethics and Medical Humanities Northwestern University Feinberg School of Medicine Chicago IL USA; ^5^ Kovler Organ Transplant Center Northwestern University Feinberg School of Medicine Chicago IL USA; ^6^ Department of Communication Studies Northwestern University Chicago IL USA

**Keywords:** consolidated framework for implementation research, health disparities, healthcare administrator, Hispanic/Latinx, implementation science, living kidney donation

## Abstract

**Background:**

Despite available evidence‐based interventions that decrease health disparities, these interventions are often not implemented. Northwestern Medicine's^®^ Hispanic Kidney Transplant Program (HKTP) is a culturally and linguistically competent intervention designed to reduce disparities in living donor kidney transplantation (LDKT) among Hispanics/Latinos. The HKTP was introduced in two transplant programs in 2016 to evaluate its effectiveness.

**Objective:**

This study assessed barriers and facilitators to HKTP implementation preparation.

**Methods:**

Interviews and group discussions were conducted with transplant stakeholders (ie administrators, nurses, physicians) during implementation preparation. The Consolidated Framework for Implementation Research (CFIR) guided interview design and qualitative analysis.

**Results:**

Forty‐four stakeholders participated in 24 interviews and/or 27 group discussions. New factors, not found in previous implementation preparation research in health‐care settings, emerged as facilitators and barriers to the implementation of culturally competent care. Implementation facilitators included: stakeholders’ focus on a moral imperative to implement the HKTP, personal motivations related to their Hispanic heritage, and perceptions of Hispanic patients’ transplant education needs. Implementation barriers included: stakeholders’ perceptions that Hispanics’ health insurance payer mix would negatively impact revenue, a lack of knowledge about LDKT disparities and patient data disaggregated by ethnicity/race, and a perception that the family discussion component was immoral because of the possibility of coercion.

**Discussion and Conclusions:**

Our study identified novel barriers and facilitators to the implementation preparation of a culturally competent care intervention. Healthcare administrators can facilitate organizations’ implementation of culturally competent care interventions by understanding factors challenging care delivery processes and raising clinical team awareness of disparities in LDKT.

## 1 INTRODUCTION

Health disparities persist as a significant public health problem,^1^ despite the availability of effective evidence‐based interventions,^2^ including culturally competent and adapted interventions,^3^, ^4^ because these interventions are not widely implemented or not delivered as intended (ie with fidelity). For example, ethnic/racial disparities in access to living donor kidney transplantation (LDKT) have increased in the last decade.^5^ Hispanics/Latinxs waitlisted for kidney transplant received significantly fewer LDKTs than waitlisted non‐Hispanic Whites in 2019: 5.0% versus 12.2%.^6^ Because LDKT offers longer kidney graft and patient survival than deceased donor kidney transplantation,^7^ LDKT disparities may magnify ethnic/racial disparities in transplant outcomes.^8^

Northwestern Medicine's^®^ Hispanic Kidney Transplant Program (HKTP) was established in 2006 to provide culturally competent and linguistically congruent care to Hispanic/Latinx patients and their families seeking evaluation for kidney transplantation. Cultural competency refers to: ‘A set of values, principles, behaviours, attitudes, policies and structures that enable organizations and individuals to work effectively in cross‐cultural situations’.^9^ The HKTP addresses recipient‐donor, health‐care provider, and health system factors known to contribute to lower rates of LDKT in ethnic/racial groups,^10^ including lack of knowledge, cultural and religious beliefs about transplantation, lack of bilingual staff at dialysis facilities and transplant programmes, and lack of culturally competent care.^11^ The HKTP was associated with a 74% increase of Hispanics receiving LDKTs and 70% decrease in the proportion of Hispanic LDKTs to non‐Hispanic white LDKTs.^12^

One factor contributing to ongoing disparities is that organizations often encounter considerable barriers to carrying out interventions.^13^ Although many culturally competent care interventions have been put into effect across clinical conditions,^3^ and some have evaluated the barriers and facilitators to their implementation, few have directly evaluated their implementation and/or used an implementation science theoretical framework to guide their implementation evaluation.^14^, ^15^ Such trends have been attributed, in part, to the implicit focus of equity in implementation and dissemination research.^16^ Further, few interventions are multilevel, that is, directed at more than patient, provider, system, social, policy or environmental levels of influence on health necessary for reducing health disparities.^17^ Moreover, few interventions aim to change clinical microsystems,^18^, ^19^ or small groups of people who routinely work together to provide health care to patients.^2^ Thus, little is known about how system factors (eg health‐care teams, hospitals, health systems) affect implementation of interventions to reduce racial/ethnic health disparities in access to care.^18^, ^20^

The purpose of this study was to identify the facilitators and barriers of HKTP pre‐implementation. Implementation research scholars recommend evaluating the barriers and facilitators to putting the intervention into effect in the preparation phase to ensure the validity of the observations.^21^ ‘Implementation preparation’ (or ‘pre‐implementation’) research occurs after an organization's leadership has decided to adopt an intervention but before it is carried out.^22^ The preparation phase also includes undertaking ‘implementation strategies’, which are ‘methods or techniques used to enhance the adoption, implementation and sustainability of a clinical programme or practice’,^23^ such as training stakeholders about the intervention and further assessing organizational needs for adaptation. The preparation phase is valuable for increasing intervention adoption and fidelity.^24^, ^25^

Implementation research examines ‘methods to promote the systematic uptake of research findings and other evidence‐based practices into routine practice, and, hence, to improve the quality and effectiveness of health services’.^26^ Implementation research aims to shed light on the gap between expected outcomes based on scientific and clinical evidence, and outcomes experienced by healthcare organizations in their implementation of those recommendations.

The Consolidated Framework for Implementation Research (CFIR)^27^ guided the study's implementation design and evaluation.^11^ CFIR is a meta‐theoretical framework compiled from 19 frameworks; it includes 39 constructs in 5 domains—intervention characteristics, organizational inner setting, characteristics of individuals, outer setting, and process.^21^ CFIR can be used as a data collection or analysis tool in any stage of the implementation (eg preparation, executing, reflecting).

Few studies to date have used CFIR (or other implementation science frameworks) to examine barriers and facilitators to interventions in healthcare settings during the preparation stage.^14^, ^21^ Intervention characteristics identified as facilitators include the following: strength of evidence^28^ and relative advantage over existing practice,^29^, ^30^ adaptability,^28^ trialability^28^, ^29^ and design quality and packaging.^31^ The primary intervention characteristic barrier found in prior research is complexity.^28^, ^29^, ^32^ Outer setting characteristics enabling intervention implementation in healthcare settings include the following: relationships between the healthcare organization and other organizations^31^ and the presence of best practice examples in other healthcare organizations.^28^ No outer setting barriers have been identified in CFIR research in the preparation phase. Inner setting characteristics identified as facilitators in healthcare settings include the following: readiness for the implementation,^30^ particularly having sufficient resources to implement the intervention,^29^ the nature and quality of teamwork^31^ and communicated leadership commitment.^28^ Inner setting characteristics identified as barriers to interventions were: competing organizational priorities^29^, ^31^ and perceived lack of compatibility with existing work routines and technology systems.^32^ Characteristics of individuals identified as facilitators in healthcare settings include the following: knowledge and beliefs about the intervention,^28^ self‐efficacy^29^ and a sense of belonging among the staff.^31^ Individual characteristic barriers include the following: resistance to new routines,^28^ limited knowledge or negative attitudes about the intervention,^30^, ^32^, ^33^ and turnover.^29^ The only process factor identified as a facilitator was a stepwise rollout.^28^ A systematic review of interventions that used no or other theoretical frameworks identified an additional barrier not mentioned in CFIR research: safety/legal and ethical concerns in the context of patient confidentiality, legal restrictions, and fear of litigation.^34^

Although prior research revealed common facilitators and barriers to implementing interventions to improve health outcomes across the population in healthcare settings, unique facilitators and barriers may arise upon implementing culturally competent care interventions designed to reduce racial/ethnic inequity. Many studies have examined facilitators and barriers to the implementation of culturally competent care interventions.^14^, ^15^, ^35^, ^36^, ^37^, ^38^ However, few of these studies highlight the unique factors relating to the implementation of culturally competent care, and/or use implementation science theoretical frameworks or models to guide study design and/or situate findings within implementation research.^14^, ^15^ Unique facilitators identified included the following: a recognition of the changing demographics of the client population, the medical center's explicit commitment to diversity, past experience with multicultural interventions, and a shared commitment to serve underserved populations. However, the barriers to implementing culturally competent care were consistent with those in previous research on other types of interventions in healthcare organizations.

## 2 METHODS

### 2.1 The culturally competent transplant care intervention

**FIGURE 1:**
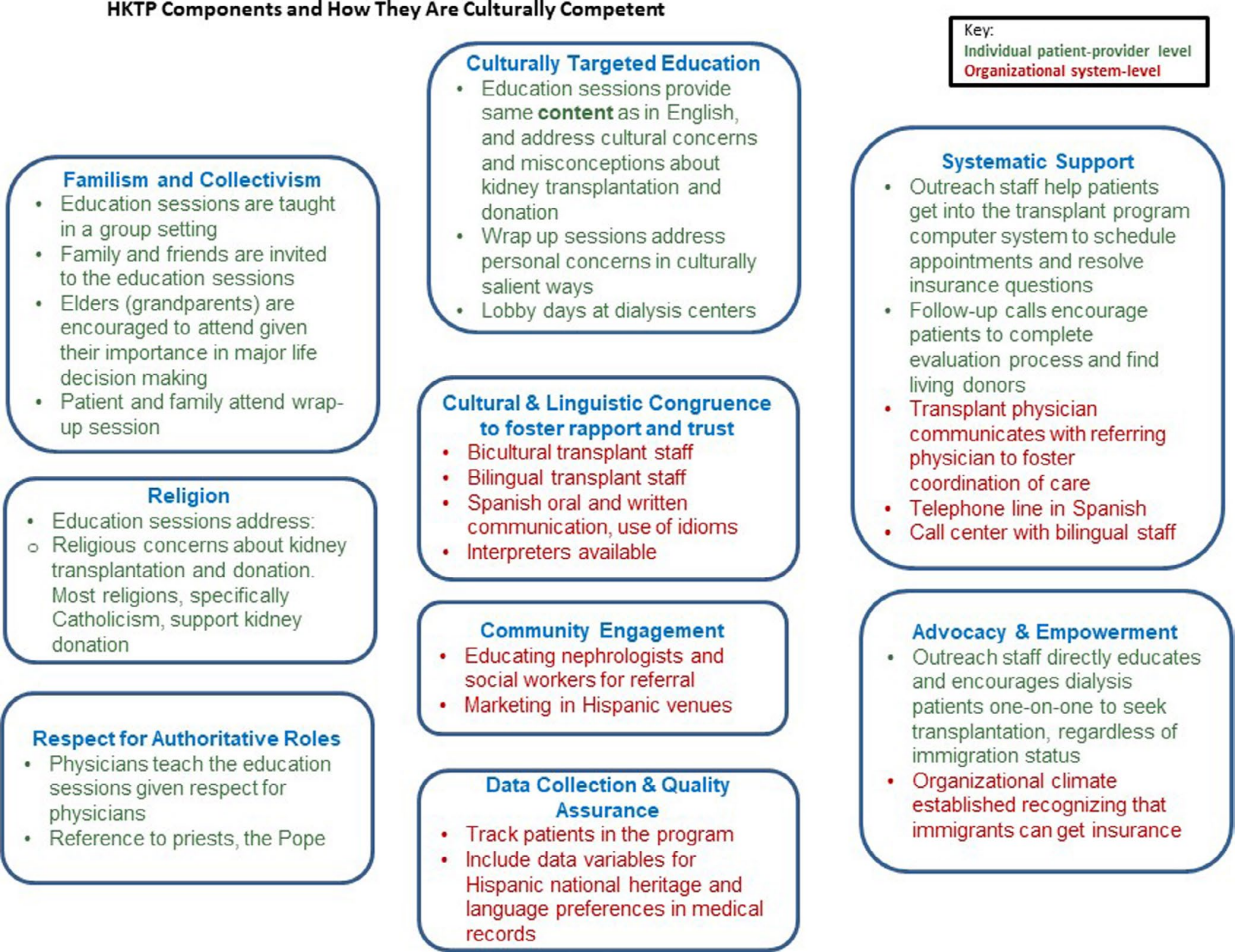
HKTP components and how they are culturally competent

The HKTP provides patients care under the same *standards* but utilizes different *care delivery processes* compared with patients receiving transplant education and evaluation in English. The HKTP intervention entails 16 key components, which map directly to the National Quality Forum's Framework for Measuring and Reporting Cultural Competency, as described^11^, ^12^ (Figure 1). Outreach to Hispanic dialysis patients about the HKTP aims to educate patients about transplantation and encourage them to seek transplant evaluation at the institution. The education sessions for potential transplant recipients and their family and friends covering routine transplant information is supplemented by culturally salient information targeted to Hispanics. A physician teaches the education sessions because many Hispanics/Latinos regard physicians as authority figures. A post‐education ‘wrap‐up’ session with the physician educator, patient and family are intended to foster family‐wide discussion and decision making about the option of and eligibility for living donation. Family members, especially elders, are encouraged to partake in the evaluation process given traditional roles in health decision making. Bicultural staff are essential to foster trust and rapport with patients through shared cultural idioms. As a linguistically congruent intervention, the HKTP entails oral and written communication in Spanish. The HKTP maintains both the potential donor's and potential recipient's confidentiality.

### 2.2 Study design and research sites

An ethnographic, longitudinal approach assessed shared perceptions of barriers and facilitators to the HKTP implementation preparation period. Utilizing an ethnographic approach entailed: examining barriers and facilitators within their social, economic, and political contexts, revealing culturally embedded norms and tacit assumptions shared among stakeholders, and examining social processes in greater depth.^39^ Northwestern University's Institutional Review Board granted study approval (STU00201331) before data collection. Written and verbal informed consent were obtained for individual interviews and group discussions, respectively.

The intervention was implemented at two US kidney transplant programs: in the South (Site A) and in the Southwest (Site B). These sites were selected because they perform 50 + living donor kidney transplants per year, have a Hispanic, bilingual transplant physician and serve a large Hispanic patient population. Both sites had disparities in LDKT rates for Hispanics compared with Whites in 2016.^12^ Both hospitals were non‐profit. Site A was a regional based academic affiliated medical center that had a large‐sized 1,000‐bed hospital with a level one trauma center. Site B is part of a national academic medical center that had a medium‐sized 300‐bed hospital with no trauma center. Implementation preparation spanned from April 2016 to December 2016 to prepare for delivering the HKTP intervention in January 2017. Although the sites were familiar with one Principal Investigator through their joint membership and participation in the American Society of Transplant Surgeons, they had no financial or collaborative relationships with the research team prior to the grant‐funded study.

### 2.3 Participants and sampling strategy

Eligible participants included transplant stakeholders: transplant physicians (surgeons, nephrologists, urologist), administrators, clinical staff involved in HKTP preparation and future implementation and research staff. Site Principal Investigators notified all stakeholders who would be directly or indirectly involved in implementing the HKTP about the forthcoming site visit and requested their participation in interviews and group discussions to facilitate the implementation process.

### 2.4 Data collection

The study Co‐Principal Investigators (EJG, JCC) conducted site visits in May 2016 to identify stakeholder, operational, and center‐level barriers and facilitators to HKTP implementation; clarify the protocol; and troubleshoot ways to accommodate the intervention into each institutional setting. Site Principal Investigators recruited stakeholders for the initial group meeting.

In‐person group unstructured discussions were led by the Co‐Principal Investigators to clarify the study protocol, assess progress on intervention implementation preparation strategies using a checklist and brainstorm ways to accommodate the intervention. In‐depth semi‐structured interviews were conducted with transplant stakeholders in person or by telephone by one Co‐Principal Investigator, a trained social scientist (EJG). Interviews assessed stakeholders’ perceptions of organizational readiness to change, organizational culture, attitudes about the implementation complexity, and perceived barriers and facilitators to implementing the HKTP components using the CFIR Interview Guide (www.cfirguide.org). Interviews lasted 30‐60 minutes and were audio‐recorded.

The learning collaborative method was used to help both sites’ transplant stakeholders design center‐customized solutions to barriers to implementing the HKTP. This rapid approach to health‐care quality improvement is used by organizations and providers to accelerate learning by collaboratively sharing their experiences and best practices.^40^

The first learning collaborative discussion occurred via a one‐hour teleconference call in September 2016. The second learning collaborative occurred during a two‐day in‐person meeting at Northwestern University in October 2016. On both occasions, stakeholders discussed challenges and strategized solutions for implementing the HKTP at their institutions, following a meeting agenda listing key intervention components for review. Half‐way through the in‐person meeting, subgroup breakout discussions were held among 2‐5 stakeholders with common roles (eg administrators, clinicians, outreach staff) to help stakeholders identify role‐specific potential roadblocks and brainstorm ways to implement the HKTP within their institutional context. Discussions were recorded mostly by audio, or handwritten notes when requested, and lasted 20‐190 minutes.

### 2.5 Data analysis

Audio‐recordings were transcribed, and transcriptions were analysed for themes using the constant comparative,^41^ deductive and inductive coding methods.^42^ Research team members with expertise in implementation science (MS, EJG) developed an initial deductive code list a priori, based on the CFIR framework domains. Each interview transcript was independently coded by the 4‐member research team. The research team held analytic retreats to review coded transcripts to inductively develop additional codes and revise the codebook for clarity, in an iterative process, until reaching data saturation (when no new information or themes emerged).^42^ Each transcript was then coded independently by two different people. After consistently achieving inter‐rater reliability on a subset of transcripts (Kappa > 0.80), all transcripts were recoded and Kappas calculated. Discrepancies between coders were resolved through arbitration by one team member (EJG). Recoded transcripts were uploaded into qualitative analysis software (MAXQDA v.12). Table 1 lists the codes by CFIR domain.

**TABLE 1:** Codes and Frequencies by CFIR Domains

Code	Frequencies	Themes—CFIR Domains
Compatibility	168	Intervention Characteristics
Infrastructure	96	Inner Context
Leadership Support	70	Inner Context
Available Resources	52	Inner Context
Team Culture	42	Inner Context
Support	41	Characteristics of Individuals
Relative Advantage	28	Intervention Characteristics
Confidence	27	Characteristics of Individuals
Institution's or Professional's Espoused Values	27	Inner Context
Business Case	24	Emergent factors related to culturally competent care (Outer Context)
Patient Needs	23	Outer Context
Evaluation and Feedback Process	20	Inner Context
Stage of Implementation	18	Implementation Process
Spanish‐Speaking Staff	16	Inner Context
Competitive Advantage	14	Intervention Characteristics
Change Commitment	14	Characteristics of Individuals
Hispanics	12	Emergent factors related to culturally competent care (Outer Context)
Immorality	11	Emergent factors related to culturally competent care (Outer Context)
Rationale	11	Emergent factors related to culturally competent care (Characteristics of Individuals)
Data Ignorance	11	Emergent factors related to culturally competent care (Inner Context)
Equity	10	Emergent factors related to culturally competent care (Intervention Characteristics)
Evaluation and Feedback Process	9	Inner Context
Learning from Past Experiences	9	Inner Context
Organizational Priorities	6	Inner Context
Evidence quality & strength	4	Intervention Characteristics
Cosmopolitan	3	Outer Context
Adaptability	1	Intervention Characteristics
Complexity	0	Intervention Characteristics

Text segments for each code were then independently reviewed by two team members to create a code summary. Each summary was developed by comparing segments and grouping together similar ideas to identify emergent patterns and themes, and comparing themes in one code summary to themes in other code summaries.^43^ We also compared and contrasted themes by study sites to see if codes were common to both sites or idiosyncratic to one site, we focused our analysis on themes common to both sites. Next, the research team reviewed all codes to see whether they mapped to CFIR; codes that did not map onto CFIR revealed new factors related to the implementation preparation of culturally competent care interventions. The research team met in groups of 2‐4 people to review each Discussion transcription and create a new document that summarized key points extracted from the discussion pertaining to relevant codes. Representative quotations are presented below to illustrate results. Credibility and confirmability of interpretations were attained by employing analyst triangulation and self‐reflexivity, and dependability (reliability) and transferability were maintained through an audit trail of analytic decisions.^44^, ^45^

## 3 RESULTS

### 3.1 Characteristics of the sample

Forty‐four stakeholders (site A: n = 21, site B: n = 23; 100% recruitment rate) participated in one or more of the following activities: a site visit interview (n = 24), group discussion (n = 35) and/or learning collaborative discussion (n = 12). During site visits, 27 group discussions occurred (site A: n = 15, site B: n = 12). Stakeholder members in group discussions varied depending on topic covered (range: 1‐9). Most participants were female (57%, site A: 52%, site B: 61%) and non‐Hispanic (80%, site A: 76%, site B: 83%) and included physicians (25%, site A: 28%, site B: 22%), nurses, social workers (30%, site A: 14%, site B: 43%), administrators (16%, site A: 19%, site B: 13%) and other staff (eg marketing, information technologists, financial and research) (30%, site A: 38%, site B: 22%).

### 3.2 Common health‐care organization factors influencing HKTP implementation

The HKTP intervention encountered facilitators and barriers common to research on implementing interventions into healthcare settings.^34^ These results correspond to two CFIR domains: intervention characteristics and the inner setting. Illustrative, representative quotations documenting these results are presented below and in greater depth in the Appendix 1.

#### 3.2.1 Intervention characteristics

Stakeholders at both sites reported that they perceived that the HKTP would benefit their current transplant program by increasing Hispanic LDKT rates and improving their program's quality of care. Thus, they perceived that the intervention had a relative advantage over existing routines. Stakeholders said that the HKTP would ‘enhance’ their organization by ‘providing better care’ specifically, as one nurse noted, by ‘bring[ing] in a culture of inclusion and diversity and cultural sensitivity… that meet[s] the needs of our patients with the demographic here’. They observed that bicultural/bilingual providers cultivate a welcoming and supportive environment that fosters genuine connections between patients and providers, characterized as ‘comfort’, ‘bonding’ and ‘engaged’.

Stakeholders commonly expressed that the HKTP held the potential to increase transplant program revenues. They were ‘motivated’ to support the HKTP as a ‘valuable endeavour’ because it aimed to increase patient volume by increasing LDKTs, which they perceived would financially benefit the institution by providing a higher revenue margin (cost versus reimbursement) than deceased donor kidney transplants:

I think there is an obvious economic benefit to [site B], I mean, more live donor transplants is economically beneficial to the transplant programs, and even though it takes resources to build a process like this, … there’s much more to gain from it than the actual investment of personnel and time… (B11)

#### 3.2.2 Inner setting

Stakeholders at both sites were receptive to the HKTP because their institutions’ values of ‘the patient comes first’, ‘compassion, integrity, respect, diversity’ and ‘people, service, [and] quality’ directly ‘aligned’ with the HKTP’s goal of providing culturally competent and linguistically congruent care. Stakeholders perceived the HKTP as ‘something different’ that had not been done before. Thus, they believed the HKTP would advance their institution's value of innovation by providing a service that ‘nobody else has’ and that ‘sets [them] apart’.

Both transplant programs’ institutional infrastructure presented challenges to implementing the HKTP. Because patient education had traditionally been the role of nurses, stakeholders expressed concern about physicians’ knowledge and skill in teaching. Some stakeholders feared that the surgeons would not have time to deliver education sessions because ‘surgeries have to come first’ in clinical care. Moreover, they worried that using surgeon time for education would be cost prohibitive.

Although both sites had abundant interpreters, sites differed in making Spanish‐speaking staff available to support the HTKP. Both sites reported lacking Spanish‐speaking staff to perform outreach at dialysis centers, assist with clinical assessments and clinical follow‐up. During the site visit, the Co‐Principal Investigators asked stakeholders to identify bilingual transplant faculty, staff, and administrators for involvement in intervention implementation. Stakeholders were initially unaware of who their bilingual staff were, but later unexpectedly learned that some staff were bilingual. The Co‐Principal Investigators suggested reallocating Spanish‐speaking staff from other departments to support the HKTP. However, stakeholders believed this option would complicate financial systems, and bilingual staff would not have time for the HKTP because they were already overworked.

### 3.3 Tensions over implementing the culturally competent care intervention

New factors, not commonly found to influence the implementation preparation of interventions in healthcare settings, arose in the HKTP implementation preparation phase.

#### 3.3.1 Facilitator 1: Equity

Stakeholders perceived the HKTP as the morally ‘right thing to do’ and appreciated how the HKTP enabled their institutions to provide equitable care to the Hispanic population. Stakeholders recognized that the Hispanic community comprises an underserved population and considered increasing services to this population important. One stakeholder stated:

[A]nybody who is a healthcare provider wants to make sure, I believe, that the patient population is able to receive healthcare in an equitable way, and certainly assist with that…. I can tell you that for us, we want to do the right thing, and if there are patients out there who aren’t able to access health care because we’re just not mindful of that, then this is the right thing to do. (A10)

#### 3.3.2 Facilitator 2: Personal motivations to implement a program for Hispanics

Personal experiences motivated many stakeholders to implement the HKTP. Hispanic stakeholders, aware of Hispanic community needs, expressed their personal desire and passion to help Hispanics and increase Hispanic access to transplantation, noting, ‘Our people need this!’ Non‐Hispanic stakeholders reported their desire to conduct research to reduce health disparities, assist underserved communities, provide linguistic congruence, and reduce challenges with interpreters. One Hispanic stakeholder stated:

I see a population that’s underserved in a state where there’s so many Hispanics, maybe because they are Hispanic. I feel passionate about who I am and where I come from, and my Latin people, are Hispanics, I think there’s some need and there’s room for growth there and improvement, and how we service the population. (B22)

#### 3.3.3 Facilitator 3: Characterizations of Hispanics

Some stakeholders’ characterizations of Hispanics as a cultural group reinforced their preparation for implementing the HKTP. Specifically, two stakeholders noted that Hispanic families, particularly elders, were important in healthcare decision making. They also perceived that Hispanics had low health literacy, especially about transplant options. Each characterization enhanced positive evaluations of HKTP components including: (a) encouraging family member involvement in the education sessions to address Hispanics’ cultural needs, (b) providing education that addresses concerns held by the Hispanic community about transplantation, and (c) involving bilingual and bicultural staff with whom Hispanic patients could identify. A stakeholder reported:

Hispanics have a very strong family orientation that, for the most part, it certainly doesn’t exist in the same way among the Caucasian population, nor in the African American population. So, it’s a distinct population that works and functions differently. …there are clearly, better ways that we can approach them. (A12)

#### 3.3.4 Barrier 1: Business case—Hispanic payer mix

A few stakeholders expressed ‘reservations’ about implementing the HKTP because of the perception that increasing Hispanic patient volumes may have a negative impact on reimbursement. Stakeholders believed that the Hispanic patient population's payer mix was comprised predominantly of Medicare and/or Medicaid rather than commercial insurance. Thus, increasing Hispanic patient volume would increase the volume of Medicaid or Medicare payers, which do not reimburse as well as commercial payers and in some cases, the expected reimbursement does not cover the institution's costs. One stakeholder stated:

If you look at every transplant that you’re doing and you’re losing money on it, adding volume doesn’t help anything. … It’s not all about the money ‐ I don’t want to make that sound like that’s what we’re looking at. But if you don’t look at it, you will lose your program because then you go under. (A10)

A few stakeholders also expressed concern about potential negative financial impact by transplanting undocumented immigrants without insurance coverage. One stakeholder recalled a past challenge that occurred when undocumented patients had initiated evaluation believing that they had Medicare coverage, but they found out after transplantation that insurance did not cover the patient. A stakeholder remarked how Hispanic undocumented patients will be declined for transplantation because of an anti‐immigrant political sentiment:

[S]o we have patients that… appear to have Medicare. But as soon as they find out that they weren’t eligible for it, because they were undocumented, we didn’t get paid.…[W]e are a private not‐for‐profit, we do a lot of charity but transplant is not the charity that our program, that our healthcare system provides. And so, I think there’s some anxiety around that for us too. (A11)

#### 3.3.5 Barrier 2: Lack of knowledge about disparities

Stakeholders were generally aware of their center's patient volume data and, less so, payer mix. However, almost all lacked knowledge of their center's patient volume and outcomes data by ethnic/racial background. One stakeholder said: ‘I really don't know [how big the need to implement this initiative at my institution was], I just know we have an unmet need, and the stats you showed this morning were very telling, and actually, I was just quite taken back by it. I had not understood that it was that bad’ (A12).

#### 3.3.6 Barrier 3: Immorality

A less common barrier to implementing the HKTP post‐education ‘wrap‐up’ component was the perception that it would violate potential recipient and donor confidentiality and potentially lead to donor coercion. One stated, ‘I think that's a general question of engaging the recipients on donor issues. We are not supposed to do that. We don't do that. And that can be, there is a tension there probably between that and… the Hispanic family’. (B11) Stakeholders feared that by asking potential recipients about the number of potential donors they have, the surgeon would inadvertently place undue influence on family members present to volunteer to become donors. Site B stakeholders also feared that the surgeon calling to inform potential recipients about the number of ruled out potential donors would unduly influence patients to remind remaining potential donors to undergo evaluation. Involving the family/potential donors in patient–physician discussions may have triggered concerns about compliance with Centers for Medicare and Medicaid Services regulations mandating that potential living donors undergo a medical and psychosocial evaluation independent of the potential recipient evaluation.^46^ As one stakeholder recognized, the HKTP intentionally involves the family in treatment decision making, but misinterpreted that the HIPAA regulations, which emphasize the privacy rights of the individual patient, might interfere with involving the family.

A few stakeholders voiced another moral concern that HKTP’s focus on Hispanics could compromise patient care for non‐Hispanics. They feared that the HKTP could create ‘resentment’ among other minority patients who do not have a culturally competent program catered to their needs.

## 4 DISCUSSION

### 4.1 Summary

Our study advances knowledge of the implementation of culturally competent care interventions, and thereby extends the field of implementation research. Our study is consistent with other studies using CFIR to guide study design, but is relatively novel in assessing providers’ barriers and facilitators to intervention implementation *during the preparation phase* to explain adaptations in the implementation period.^21^ Most studies of culturally competent interventions examine intervention efficacy, but not its implementation. Our study contributes to scant research^14^, ^15^ using implementation science to evaluate the barriers and facilitators to the implementation of a culturally competent care intervention. Unlike many culturally competent interventions that focus on the patient‐provider interaction, our study intervened on multiple‐levels beyond the patient‐provider interaction (ie outreach, marketing, clinic education, scheduling processes). Thus, we identified a more holistic set of barriers and facilitators involved in implementing a culturally competent care intervention, thereby advancing implementation research designed to increase health equity.^16^ We identified several novel facilitators and barriers to the implementation preparation of a culturally competent care intervention targeting the Hispanic patient population not identified in previous CFIR research on healthcare settings.

### 4.2 Comparison with existing literature

Some of these themes are consistent with research directly evaluating barriers and facilitators to the implementation of culturally competent care in healthcare settings.^14^, ^15^ Specifically, healthcare settings’ institutional values of patient‐centeredness and commitment to diversity were seen as consistent with HKTP’s goal of providing culturally and linguistically competent care. Moreover, in both Nagy's and our studies, client demographics were seen as a motivator of the intervention. Similarly, Black patients’ perception that the US Department of Veterans Affairs genuinely wanted to help them was classified as a facilitator of the intervention.^14^

However, we identified several novel facilitators and barriers related to the intervention's culturally competent character, specifically, its focus on addressing Hispanics. Facilitators included stakeholders’ sense of moral authority to address institutional health disparities, perceptions that the intervention can address Hispanics’ cultural needs and personal motivation as members of the target population to help Hispanic patients. Each of these additional individual characteristics, not identified in CFIR implementation preparation research, influences receptivity to culturally competent care interventions targeted to Hispanics. Healthcare administrators involved in implementing culturally competent care interventions should recognize the value of stakeholders who share not only language, but also culture with the targeted population to champion the intervention. Administrators may consider reallocating bilingual staff from different departments. Administrators should convey to stakeholders the ethical value of the intervention to foster effective implementation.

However, barriers included stakeholders’ concern about the negative financial impact of increasing the number of Hispanic patients due to their Medicaid or undocumented status and stereotypes about Hispanics. Both factors would be part of the outer setting in the CFIR framework, if factual. However, these factors reflect a lack of knowledge, an individual characteristic. The knowledge gap was not about the intervention, but about Hispanics. Stakeholders at site A believed that Hispanics’ payer mix was heavily government subsidized. However, in 2016, more Hispanic transplant recipients at site A had commercial insurance than non‐Hispanic white recipients by 10%, and more non‐Hispanic white transplant recipients had government‐subsidized insurance (ie Medicaid and/or Medicare) than Hispanic recipients by 10%.^47^ Nationally, 40% of Hispanic adults have employer or commercial health insurance.^48^ Our findings suggest some stakeholders held implicit bias about Hispanics. Implicit bias can influence provider behaviors and patient outcomes.^49^ Considerations over financing of the healthcare system similarly emerged as a barrier to a mobile health application intervention in Kenya and Canada.^38^

Another barrier was stakeholders’ lack of awareness of and the absence of data on LDKT rates for Hispanics compared with non‐Hispanic whites at both sites. A national survey also found that most dialysis providers were unaware of racial/ethnic disparities in transplant wait‐listing (81%), and within their own dialysis facility (95%).^50^ Conversely, in other industries (ie education), data are commonly disaggregated by race/ethnicity to look for disparities.^51^ Collecting and reviewing outcomes by ethnic/racial groups are necessary for identifying the presence of disparities, measuring their magnitude, and setting institutional goals towards increasing equity, as recommended by The Robert Wood Johnson Foundation's Finding Answers initiative for reducing health disparities.^18^ Transplant centers are mandated to report patients’ demographics, including ethnicity and race, to OPTN/UNOS. However, our finding suggests that sites did not evaluate or were not cognizant of those data.

The HKTP comprised a challenge to study sites’ current care delivery processes by using physicians as educators, involving family (and potential donors) in potential recipients’ decision making and using bicultural staff. As such, it is a complex intervention that targets several different healthcare organization processes (eg scheduling, job design, human resources). Stakeholders perceived HKTP’s culturally competent care through the lens of reasonable accommodation. That is, they viewed cultural and language differences as barriers to receiving the current care delivery processes, not recognizing that the same current care delivery processes offered to all patients produced less favourable outcomes for ethnic minority patients.

Although stakeholders were informed about how HKTP components were culturally competent and the value of culturally competent care, our results suggest that some stakeholders did not seem to fully grasp the meaning of culturally competent care. Instead, many viewed the HKTP as a way of overcoming the perceived additional needs of Hispanic patients, and primarily construed the HKTP as providing the same form of care, but in Spanish. Furthermore, stakeholders did not question the implicit ways that their institution's current care delivery process prioritized non‐Hispanic white cultural values.

Anthropologically, tacit cultural values of individualism, health‐seeking behaviors, and nosology, embedded within US health‐care practice reinforce an individual‐centered rather than family‐centered approach to patient care.^52^, ^53^ Healthcare administrators involved in culturally competent care interventions should consider having stakeholders undergo training in cultural competency and to identify implicit biases. Such training may help stakeholders become more receptive to alternative care delivery processes that prioritize the needs of underserved patients.

By maintaining one care delivery process as the gold standard that all patients receive, hospitals will inevitably impede the provision of culturally competent care, and the reduction of health‐care disparities.^11^, ^18^ Culturally competent care requires healthcare organizations to adopt different care delivery processes, rather than rely on a singular care delivery process for all patients. Some stakeholders believed that delivering culturally competent care for one ethnic minority group would compromise care for other groups. Their underlying assumption was that fairness means that all patients receive the same health‐care resources (equality). Instead, culturally competent care aims to promote equity, which means that all patients receive the care they need to achieve the same healthcare results.^54^ We recommend that healthcare leaders understand and articulate to their teams that institutions should strive for equity in outcomes, as opposed to equality in care delivery processes, to foster culturally competent care.

### 4.3 Implications for research and clinical practice

We recommend several strategies to facilitate the implementation of the HKTP or other culturally competent care interventions in healthcare institutions (Table 2). During the implementation preparation phase, transplant healthcare administrators should leverage their knowledge of hospital operations and access to patient‐level data to identify optimal ways to accommodate the intervention within the institution, and conversely, to adjust the institutional infrastructure to accommodate the intervention. Accordingly, they should plan for and monitor costs and reimbursements associated with the intervention. Healthcare leadership should also analyze patient outcomes data by racial/ethnic groups to identify potential disparities and use these data to set program goals for improving these outcomes and increasing ethnic/racial equity. Healthcare administrators should educate stakeholders often about disparities in transplant access and outcomes in their patient population, to prime them to deliver different care delivery processes embedded in culturally competent care. Transformative leadership styles that mobilize team understanding of an intervention can foster organizational climates conducive to implementing culturally competent care interventions.^55^ Further, leadership should educate stakeholders about tacit cultural assumptions underlying the existing care delivery processes to avert expressions of subtle cultural biases and accommodate the needs of underserved populations. Moreover, leadership should proactively champion the business case that culturally competent care interventions advance the institutional mission of serving the community and thus are the ‘right thing to do’. Future research should evaluate how these novel constructs affect implementing other culturally competent care interventions.

**TABLE 2:** Practice Recommendations by HKTP Needs, Challenges to Implementation and Potential Solutions

Needs	Challenges to implementation	Potential solutions
Bicultural/Bilingual staff	Traditional institutional reliance on interpreters, and the belief that interpreters are ‘good enough’ Difficulty hiring bilingual/bicultural clinical staff due to the limited pool of qualified candidates Lack of understanding of the transplant team's demographics in relation to ethnic background and language skills	Recruit people who are bilingual/bicultural to fill open faculty/staff positions Re‐allocate bilingual/bicultural staff from other hospital departments Post positions year round Post positions in the *Hispanic Health Care International (HHCI)* journal, which is the official journal of the National Association of Hispanic Nurses, or other bilingual venues Assess the transplant team's ethnic background and languages spoken Leverage intervention champions of a similar ethnic background as the target patient population
Awareness of ethnic/racial disparities in transplant patient volume and outcomes	Tradition of analyzing patient transplant data in aggregate, not broken down by race/ethnicity or other demographic variables	Analyse transplant center patient volume and outcomes data by ethnicity/race and/or other groups volume and outcomes Raise clinical team awareness of disparities by posting leaflets or posters describing disparities in private areas (eg conference rooms, hallways), and discussing in staff meetings
Inaccurate assumptions to be corrected	The following inaccurate assumptions: That the Hispanic payer mix represents a disadvantageous payer mix without analysing center data That undocumented Hispanics are not able to purchase insurance coverage	Analyse center payer mix for Hispanics (and/or other groups) Help patients obtain coverage prior to starting the evaluation process Analyse center revenue on an ongoing basis Inform center stakeholders about Hispanic payer mix Inform stakeholders that undocumented Hispanics can purchase health insurance coverage (but not through health insurance exchanges which are publicly subsidized) without a social security number most of the time, according to anecdotal reports by transplant social workers. The US Internal Revenue Service (IRS) can issue an Individual Taxpayer Identification Number (ITIN), which is a tax processing number, regardless of immigration status.^56^ The IRS wants to ensure that people, including unauthorized immigrants, pay taxes even if they do not have a Social Security Number and regardless of their immigration status Emphasize the ethical value of implementing culturally competent care as a good business model
Embrace care delivery processes that foster equity, rather than equality	Misperception that different care delivery processes for different patients groups is unfair	Perform a cultural assessment of transplant stakeholders to identify intrinsic bias^57^ Educate stakeholders about cultural biases

### 4.4 Strengths and limitations

A strength of this study is that it was conducted in multiple sites, contributing to transferability of study findings. Another strength is the use of the implementation science theoretical framework, CFIR, to guide analysis of the implementation preparation process.

A study limitation is that participants’ statements or perceptions may not reflect actual behaviours. Although our results may be transferrable to academic, non‐profit hospitals, results may differ in community hospitals and/or hospitals in other US geographic regions. Study findings may reflect US experiences in implementing culturally competent care interventions highlighted by its market‐based system that may not arise in countries with a single payer system. A social desirability bias may have softened stakeholders’ concerns because grant funding supported HKTP implementation. We used measures to control for social bias including informing participants that their input would be analysed in aggregate and contribute to a better understanding of how to implement the HKTP and deliver culturally sensitive care for Hispanic patients in the future. Perceived barriers may not have prevented implementation.

## 5 CONCLUSION

Our study identified novel barriers and facilitators unique to the implementation preparation of a culturally competent care intervention that reflect implicit biases about delivering care to cultural groups. Our findings may enable healthcare organizations to more effectively implement the HKTP and other culturally competent care interventions in the future.

## CONFLICT OF INTEREST

No authors have a conflict of interest.

## AUTHOR CONTRIBUTIONS

EJ Gordon, M Shumate: Conception and design; EJ Gordon, M Shumate, E Romo, D Amortegui, A Rodas, N Anderson, J Uriarte: Analysis and interpretation of the data; EJ Gordon, M Shumate: Drafting of the article; EJ Gordon, M Shumate, E Romo, D Amortegui, A Rodas, N Anderson, J Uriarte: Critical revision of the article for important intellectual content; EJ Gordon, E Romo, D Amortegui, A Rodas, N Anderson, J Uriarte, JC Caicedo, M Shumate: Final approval of the article; EJ Gordon, JC Caicedo: Obtaining of funding; EJ Gordon, E Romo: Administrative, technical, or logistic support; EJ Gordon, JC Caicedo: Collection and assembly of data.

## Data Availability

Study protocol: Available from Elisa Gordon (e‐gordon@northwestern.edu). Qualitative data: The data are not publicly available because the participants were not asked as part of the informed consent process to give their approval for sharing data resulting from their participation, or to use their data for other research studies, and because the data set contains information that could compromise research participant and institutional privacy/confidentiality.

## Appendix 1 Representative illustrative quotations by theme

**Table:**

Facilitators	
Equity	‘[A]nybody who is a healthcare provider wants to make sure, I believe, that the patient population is able to receive healthcare in an equitable way, and certainly assist with that….I can tell you that for us, you know, we want to do the right thing and if there are patients out there who aren't able to access healthcare because we're just not mindful of that, then this is the right thing to do’. (A10)
	‘It's a great idea what you are doing. Increasing awareness about the living donation in the Hispanic population… Certainly, Hispanics as a population may be even more attractive because of the barriers that they may have ‐‐ be it, cultural, be it language barriers, so… we have the opportunity to be even more effective in this group, if we can abolish those barriers… It's time for our group to do this in a systematic fashion, so [site B] is very well positioned to get on this and get the job done. And we have a fairly good amount of the Hispanic population. I can't tell the exact percentage…. And we would like to see the living donation improve at our center…’ (B10)
	‘[S]omething like this, I think offers us the opportunity to not only focus on underserved populations but also I think will enhance or change some of our thinking related to all populations…’ (B16)
Personal motivations to implement a program for Hispanics	‘For me personally, I think it's [the HKTP] a really important one being of Hispanic, just being Hispanic myself, you know, and having parents… I mean, I understand the value of it from a personal level, and then from generally speaking for the institution, I think, it's also very beneficial, it definitely brings in a culture of inclusion and diversity and cultural sensitivity, so I think that as an institution it's very valuable as well that we are meeting the needs of our patients with the demographic here in [state]’. (B13)
	‘I think to me, I see a population that's underserved in a state where there's so many Hispanics, maybe because they are Hispanic and I feel passionate about who I am and where I come from, and my Latin people, are Hispanics, I think there's some need and there's room for growth there and improvement, and how we service the population, and I think because it's transplant and I love transplant, it was one of those things, you just do something good for somebody and they are grateful, most of them, I can't say everybody is, most of them are grateful for that second chance. So I think, for me, it's the passion and excitement, and to be able to be involved with something like that, it's great personally’. (B22)
	‘You know, as a native Spanish speaker, my abilities have been utilized since my medical school days, and I think I’ve been impactful on a patient to patient basis most of my career, and I think this is an opportunity to impact the community of potential patients on a broader scale…’ (A13)
Characterizations of Hispanics	‘I look around the community, and I’m like this [the HKTP] is obviously a huge need. And the other thing I admire a lot about the Hispanic population and Hispanic culture is they are very loyal as a culture. I think once they see somebody catering to their needs, I think it will automatically drive people to at least inquire and find out about the program. So I think it [the HKTP] has a lot of potential to grow pretty quickly just because I’ve seen the need when we opened up community events, when they come and the desire for knowledge and the questions they have and how they embrace and absorb, and they just have a thirst for that to understand it’. (A14)
	‘Hispanics have a very strong family orientation that, for the most part, it certainly doesn't exist in the same way among the Caucasian population, nor in the Afro American population. So it's a distinct population that works and functions differently, and unless you begin to just scratch at the surface and ask questions, and you see this…there are clearly, better ways that we can approach them on’. (A12)
	‘[A]nd I understand, you know, the elders and they kind of rule and dictate, but I think that if you can explain things to them, they'll be more open‐minded. I mean hopefully more open‐minded and say ‘yes’. (B14)
Business case for increasing LDKT volume	‘I think there is an obvious economic benefit to [site B], I mean, more live donor transplants is economically beneficial to the transplant programs, and even though it takes resources to build a process like this, … there's much more to gain from it than the actual investment of personnel and time….[I]f you think of it from an economic standpoint, there's much more profit here than, you know, there's going to be a lot of revenue generated by each incremental living donor transplant compared to the personnel and human time that it would take to create this program’. (B11)
	‘Well, it's… you know, certainly, with more donors becomes more recipients. So for [site B] that means more money, yeah… So, bottom line. [Giggles] I really think that, that's what motivates [site B] to want to seek this, but you know, our numbers are growing every year, especially in kidney transplant but as well as in liver but our donor population, our living donor has stagnated’. (B18)
	‘…[T]his is business as well, and it's [the HKTP] something that hopefully will grab more individuals attention to come to [site A] and get their transplants here’. (A13)
**Barriers**	
Business case and payer mix impact on revenue	‘… we have to consider the bottom line of these things. We have to think about the greater good and cost too and how to meld those things together, and we can make that happen’. (B24)
	‘Yeah, it sounds like the only reservation they [transplant department leaders] have is our payer mix, and their ability to come here for insurance. But otherwise, other than that, they think that culturally that they would adapt, and it sounds like from the past clinical research that they are very accepting and very accountable to that consent and follow through’. (B23)
	‘Well, I mean, if you look at every transplant that you're doing and you're losing money on it, adding volume doesn't help anything, so you just have to, if you're losing money then you are not at your sweet spot, and it's not all about the money I don't want to make that sound like that's what we're looking at, but if you don't look at it, you will lose your program because then you go under’. (A10)
	‘[S]o we have patients that have applied and received Medicare under the [unclear; 0:46:27] but who didn't qualify and so, they appear to have Medicare. But as soon as they find out that they weren't eligible for it, because they were undocumented, we didn't get paid.…[W]e have done a number of transplants that way and so, some of those are the fears…do we have the processes in place to ensure…but then we find out later…and we don't expect a Medicaid plan prima[rily]…we don't accept a lot of exchange plans, so we are a private not‐for‐profit, we do a lot of charity but transplant is not the charity that our program, you know, that our health care system provides. And so, you know, I think there's some anxiety around that for us too’. (A11)
Lack of Knowledge about disparities and lack of disaggregated data	‘I wasn't even really aware of the exact numbers until, you guys, brought it to our attention. I’m sure some of the transplant people knew like our whoever the nephrologist that you guys have been… I don't know who you're dealing with if it's… Dr [Department chairs] or them. They probably are aware of that, our coordinators are. I don't even know that everyone really knew the exact numbers’. (B19)
	‘I really don't know [how big the need to implement this initiative at my institution was], I just know we have an unmet need, and the stats you showed this morning were very telling, and actually, I was just quite taken back by it. I had not understood that it was that bad’. (A12)
Immorality	‘Yeah, I mean, I think that's a general question of engaging the recipients on donor issues. We are not supposed to do that, we don't do that. And that can be, there is a tension there probably between that and the family or [unclear] of the Hispanic family’. (B11)
	‘Well, the fact that they haven't come forward, I think is private, yes, there might have been a discussion in a group saying these are potentials, but any next steps by any of those donors whether it's moving forward or not, is really private and confidential to the donor, not, in my opinion is not our job to share with the recipient, donors can share that with the recipient, but we wouldn't, just in our current practice, that's just not what we speak about’. (A10)
	‘But you can't keep, you can't ask the nurses to compromise this population of patients to meet the needs of this population of patients’. (B25)
	‘It may make me wonder about sort of other minorities and wonder why we don't have a cultural sensitive program that caters to their needs too. So, I’m not sure if there's resentment from other patients who may have that, I don't know’. (B24)
CFIR Themes	
Institutional or Professional Espoused Values	‘I think the history of what we believe is always the right thing to do for the right reason, which I think everybody thinks this is the right thing to do, it's a need that we have, which is how we always decide how to do things, we looked at the needs and various to‐dos: does your patient need to do it? And, is it the best thing for the patient? I think one thing you'll see about our organization and our culture really is that we like to pride ourselves, and I think we do on being a patient‐centered organization. And because of that, it's just patient centered to a total definition of what patient centered is, right? If you do it the right way in my opinion and so to me it aligns with the core values, mission, vision and values of what [site A] stands for, and anytime we can do that, we can align anybody we need to, to get behind a project and I know that, just because everybody, that's just how we agree upon things, everything we do is around a pillar, it's people, service, quality, and finance, and if we can align our projects with those four pillars which are our core values of our culture… Then, we can move things much easier. Does that make sense?’ (A14)
	‘I mean, I think that [site B] has meant to, and I mean, as the caveat, that I’m new, but [site B] really focuses on the care of the patient, and the care of the patient comes first, and that doesn't there's not differentiation in regards to whether that patient has Hispanic background, or European background, or whatever the case may be. We are trying to provide those quality services. So I mean, by providing it, and then trying to outreach specifically to the Hispanic community we are outreaching to the population that we have here in [our state]. So, there is that great need for that and by doing this program it will really engage the local population’. (B21)
	‘The [site B] values are based on compassion, integrity, respect, diversity with those 4 values, this research project supports all of those. So, the diversity of culture, the respect of differences, and the respect of the different cultures, even within our departments of research and clinical practice, and how we are able to collaborate together to hold integrity. So, I think this supports research values and [site B] values’. (B23)
Relative advantage	‘Well, because it [the HKTP] would just bring that, it would just make that much, you know, what makes [site B] special per se or the thoroughness of it or the quality of care with the education and research backing it up, it will just bring that full range of culture and care to the Hispanic population instead of just a fraction of it, you know, by using interpreters or just having written information duplicated but not having that access to a Spanish speaking provider. So they are not getting the full experience, I guess, I would say, currently, because of that, but if they did, then it would be great’. (B13)
	‘I think that it can, yeah, I think so, I think that it's going to increase Hispanic kidney transplant and I think that in itself will increase living donor kidney transplant, so I think that's an advantage’. (A10)
Changing Roles of Physicians and Nurses	‘I will just mention this, it seemed odd to me, not odd, but I understand the concept, but having the physicians, do all the teaching, I have a little bit of anxiety with that, because I don't, I think there are things that our physicians won't know about, won't teach as well, and know, different physicians are different, there are some physicians that are excellent teachers and others are not and so, not that it can't happen, but I feel strongly, like I want to listen, participate, I feel like I need to have my hands on that a little bit more than, “Sure you just go teach!”’ (A10)
	‘I understand [DrX] does that education class, correct? [interviewer: Yes] It's, I’m concerned that we'll be able to deliver that. A little bit concerned…. but [Dr X, the surgeon educator] is very busy guy, yeah and [Dr X] is very busy. It's not just him. All of us are very busy. I’m the medical director of the program, I have just this afternoon, Wednesday afternoon is my time to do all the meetings, every time else…I’m, you know…So, this is the way it is here. It's not a bad spot. But, it is [Pause] you have to really be mindful of the resources’. (B20)
	‘…. [S]omething like this, I think offers us the opportunity to not only focus on underserved population but also I think will enhance or change some of our thinking related to all populations, as part of being able to think outside of the box. Having a physician or surgeon teach a class is so far‐fetched from anything anybody, you know, it's always, relegated to the lowest clinical provider’. (B16)
Compatibility	‘I think the biggest one [barrier] that I can personally would say is the lack of having bilingual staff, we are very limited in, there's not very many staff that's actually bilingual, I mean, we're kind of sparsed out…. I have tried the interpreters just to get a test, and a feel for them, and generally they do a very good job with interpreting information, I think it's very dependent on the interpreter. So, some interpreters are a little better than others, I guess that's a symptom of growing pains I guess, you know, if they are newer to the area or if they interpreted this material before, they are familiar with how to get the message across, but generally speaking, interpreters have been good, they have gotten the task done… But what you miss is that connection like, the trust, I guess there's a level of trust, a level of connection that you lose with an interpreter, and so that's hard to quantify and it's hard to, yeah, it's just hard to quantify and really make sense of it until you actually go through it. Yeah, so the information gets translated, the information, the questions, there's opportunity for questions, but you do lose the sense of, that intuition, you lose that sense of intuition, like do they really understand this? Do I need to explain something further? And an interpreter is just simply transplanting the words, they don't really have the intuition to know based on their responses if they are really getting it or not, so I think you do lose a little bit of that with an interpreter’. (B13)
	‘I think, because of how, to me how we're communicating with that patient population now isn't very patient centered, it's through a translator, it's through a phone, it's through a computer, which is what we use as interpreting. We don't even have a lot of one on one interpreters anymore’. (A14).
Available resources	‘Well, I know that our website needs to be translated and unfortunately I have no control over that. This is something that needs to be done by, not by [site B], it's done by [site B] enterprise. So, it's a joke, I call it the mothership. [Interviewer laughs] If we have a like 3 committees here to do something, we have about 7 to go thought the mothership, so I don't know how long is going to take but I know they are working on it. That's, that's, all I know’. (B15)
	‘For [site A], no, we can absolutely engage the social media team with our marketing group but those two teams are very trusting and love, love, love transplant stories and yet, they get a lot of attention on our social media so they're anxious for those stories. We just didn't want to launch that too soon, of course’. (A11)
Perceptions of Hispanic patients’ needs	‘…. I think the biggest thing is going to be on the pre‐transplant side of things, you know, on the [nearby site A] campus we have Spanish‐speaking coordinator, and on the [site A] campus we do not, and so, I think that's going to be a big starting point, where we need to figure out how we can, as you mentioned the other day, I mean, this patient population does not want to go through an interpreter, and they do not want to go through family members necessarily and I think that works better sometimes than an interpreter, but we need to figure that one out first’. (A15)
	‘…but I think right now we take care of people's clinical needs, but I think that for our Hispanic patients that are looking for kidney transplant education, we probably need to enhance how they are being educated because they need that and that need is real’. (B11)
	‘We have such a close relationship with the coordinators, all the coordinators because I mean we are walking with patients, we are educating them through the process, and we notice a difference between our English speaking patients and our Spanish‐speaking patients. They are less likely to call back – Spanish speaking patients. We don't get to connect with them on that level. Like today, I was talking to a patient this morning about her upcoming wedding and her trip to Bali for her honeymoon and all of these things. I would not be able to get that engaged through an interpreter, and that's what leadership doesn't see. They see us still getting the patients through and getting them transplanted and getting the outcome, the good outcomes to the SRTR, but they don't see us not being able to engage with patients’. (B17)

## References

[1] Institute of Medicine . How far have we come in reducing health disparities? Progress since 2000: Workshop summary. Washington, DC: The National Academies Press; 2012. 23193624

[2] Nelson HD , Cantor A , Wagner J , et al. Achieving health equity in preventive services: a systematic review for a national institutes of health pathways to prevention workshop. Ann Intern Med. 2020;172(4):258‐271. 31931527 10.7326/M19-3199

[3] Butler M , McCreedy E , Schwer N , et al. Improving cultural competence to reduce health disparities, Report no. 16‐EHC006‐EF. Rockville, MD: Agency for Healthcare Research and Quality (US); 2016. 27148614

[4] Escoffery C , Lebow‐Skelley E , Haardoerfer R , et al. A systematic review of adaptations of evidence‐based public health interventions globally. Implement Sci. 2018;13:125. 30257683 10.1186/s13012-018-0815-9 PMC6158804

[5] Purnell T , Luo X , Cooper L , et al. Association of race and ethnicity with live donor kidney transplantation in the United States from 1995 to 2014. J Am Med Assoc. 2018;319(1):49‐61. 10.1001/jama.2017.19152 PMC5833543 29297077

[6] United Network for Organ Sharing . Transplant trends; 2019. https://unos.org/data/transplant‐trends/ Accessed 3 January 2019

[7] National Institute of Diabetes and Digestive and Kidney Diseases . United States Renal Data System. 2019 USRDS annual data report: Epidemiology of kidney disease in the United States. Bethesda, MD: National Institutes of Health; 2019.

[8] Kwan JM , Hajjiri Z , Chen YF , Metwally A , Perkins DL , Finn PW . Donor and recipient ethnicity impacts renal graft adverse outcomes. J Racial Ethnic Health Disp. 2018;5(5):1003‐1013. 10.1007/s40615-017-0447-9 29270842

[9] Department of Health and Human Services . National Standards for Culturally and Linguistically Appropriate Services in Health Care: Final Report [www.omhrc.gov/clas/]. Rockville, MD: Department of Health and Human Services; 2001.

[10] Purnell TS , Hall YN , Boulware LE . Understanding and overcoming barriers to living kidney donation among racial and ethnic minorities in the United States. Adv Chronic Kid Dis. 2012;19(4):244‐251. 10.1053/j.ackd.2012.01.008 PMC3385991 22732044

[11] Gordon EJ , Lee J , Kang RH , et al. A complex culturally targeted intervention to reduce Hispanic disparities in living kidney donor transplantation: an effectiveness‐implementation hybrid study protocol. BMC Health Service Res. 2018;18(1):368. 10.1186/s12913-018-3151-5 PMC5956564 29769080

[12] Gordon EJ , Lee J , Kang R , et al. Hispanic/Latino disparities in living donor kidney transplantation: Role of a culturally competent transplant program. Transplant Direct. 2015;1(8):e29. 27500229 10.1097/TXD.0000000000000540 PMC4946478

[13] Morris Z , Wooding S , Grant J . The answer is 17 years, what is the question: Understanding time lags in translational research. J R Soc Med. 2011;104(12):510‐520. 22179294 10.1258/jrsm.2011.110180 PMC3241518

[14] Woodward E , Matthieu MM , Uchendu US , Rogal S , Kirchner JE . The health equity implementation framework: Proposal and preliminary study of hepatitis C virus treatment. Implement Sci. 2019;14(1):26. 30866982 10.1186/s13012-019-0861-y PMC6417278

[15] Nagy G , LeMaire K , Miller M , Howard M , Wyatt K , Zerubavel N . Development and implementation of a multicultural consultation service within an academic medical center. Cog Behav Pract. 2019;26(4):656‐675.

[16] Nooraie RY , Kwan BM , Cohn E , et al. Advancing health equity through CTSA programs: Opportunities for interaction between health equity, dissemination and implementation, and translational science. J Clin Transl Sci. 2020;4:168–175. 32695484 10.1017/cts.2020.10 PMC7348010

[17] Paskett E , Thompson B , Ammerman AS , Ortega AN , Marsteller J , Richardson D . Multilevel interventions to address health disparities show promise in improving population health. Health Aff. 2016;35(8):1429‐1434. 10.1377/hlthaff.2015.1360 PMC5553289 27503968

[18] Chin M , Clarke A , Nocon R , et al. A roadmap and best practices for organizations to reduce racial and ethnic disparities in health care. J Gen Intern Med. 2012;27(8):992‐1000. 22798211 10.1007/s11606-012-2082-9 PMC3403142

[19] O’Leary KJ , Johnson JK , Manojlovich M , Goldstein JD , Lee J , Williams MV . Redesigning systems to improve teamwork and quality for hospitalized patients (RESET): Study protocol evaluating the effect of mentored implementation to redesign clinical microsystems. BMC Health Service Res. 2019;19(1):293. 10.1186/s12913-019-4116-z PMC6505207 31068161

[20] Chinman M , Woodward E , Curran G , Hausmann L . Harnessing implementation science to increase the impact of health equity research. Med Care. 2017;5(Suppl. 92):S16‐S23. 10.1097/MLR.0000000000000769 PMC5639697 28806362

[21] Kirk MA , Kelley C , Yankey N , Birken SA , Abadie B , Damschroder L . A systematic review of the use of the Consolidated Framework for Implementation Research. Implement Sci. 2016;11(72). 10.1186/s13012-016-0437-z PMC4869309 27189233

[22] Moullin JC , Dickson KS , Stadnick NA , Rabin B , Aarons GA . Systematic review of the Exploration, Preparation, Implementation, Sustainment (EPIS) framework. Implement Sci. 2019;14(1):1. 30611302 10.1186/s13012-018-0842-6 PMC6321673

[23] Smith JD , Li DH , Hirschhorn LR , et al. Landscape of HIV implementation research funded by the National Institutes of Health: A mapping review of project abstracts. AIDS Behav. 2020;24(6):1903‐1911. 31845078 10.1007/s10461-019-02764-6 PMC7220870

[24] Koenig CJ , Abraham T , Zamora KA , et al. Pre‐implementation strategies to adapt and implement a Veteran peer coaching intervention to improve mental health treatment engagement among rural Veterans. J Rural Health. 2016;32(4):418‐428. 27509291 10.1111/jrh.12201

[25] Kowalski CP , Veeser M , Heisler M . Formative evaluation and adaptation of pre‐and early implementation of diabetes shared medical appointments to maximize sustainability and adoption. BMC Family Pract. 2018;19(1):109. 10.1186/s12875-018-0797-3 PMC6035791 29981568

[26] Eccles M , Mittman B . Welcome to implementation science. Implement Sci. 2006;1.

[27] Damschroder L , Aron D , Keith R , Kirsh S , Alexander J , Lowery J . Fostering implementation of health services research findings into practice: A consolidated framework for advancing implementation science. Implement Sci. 2009;4:50. 19664226 10.1186/1748-5908-4-50 PMC2736161

[28] Gabler G , Coenen M , Fohringer K , Trauner M , Stamm T . Towards a nationwide implementation of a standardized nutrition and dietetics terminology in clinical practice. BMC Health Service Res. 2019;19(1):920. 10.1186/s12913-019-4600-5 PMC6884883 31783855

[29] Morgan D , Kosteniuk J , O'Connell ME , et al. Barriers and facilitators to development and implementation of a rural primary health care intervention for dementia: A process evaluation. BMC Health Service Res. 2019;19(1):709. 10.1186/s12913-019-4548-5 PMC6798332 31623609

[30] Robins LS , Jackson JE , Green BB , Korngiebel D , Force RW , Baldwin LM . Barriers and facilitators to evidence‐based blood pressure control in community practice. J Am Board Family Med. 2013;26(5):539‐557. 10.3122/jabfm.2013.05.130060 PMC3882900 24004706

[31] Low LL , Ab Rahim FI , Johari MZ , et al. Assessing receptiveness to change among primary healthcare providers by adopting the consolidated framework for implementation research (CFIR). BMC Health Service Res. 2019;19(1):497. 10.1186/s12913-019-4312-x PMC6636000 31311538

[32] Hagedorn H , Wisdom JP , Gerould H , et al. Implementing alcohol use disorder pharmacotherapy in primary care settings: A qualitative analysis of provider‐identified barriers and impact on implementation outcomes. Addict Sci Clin Pract. 2019;14(1):24. 31291996 10.1186/s13722-019-0151-7 PMC6617941

[33] Folker AP , Mathiasen K , Lauridsen S , Stenderup E , Dozeman E , Folker M . Implementing internet‐delivered cognitive behavior therapy for common mental health disorders: A comparative case study of implementation challenges perceived by therapists and managers in five European internet services. Internet Intervent. 2018;11:60‐70. 10.1016/j.invent.2018.02.001 PMC6084870 30135761

[34] Geerligs L , Rankin NM , Shepherd HL , Butow P . Hospital‐based interventions: A systematic review of staff‐reported barriers and facilitators to implementation processes. Implement Sci. 2018;13(1):36. 29475440 10.1186/s13012-018-0726-9 PMC5824580

[35] Gibbs L , Waters E , Christian B , et al. Teeth tales: A community‐based child oral health promotion trial with migrant families in Australia. BMJ Open. 2015;5(6):e007321. 10.1136/bmjopen-2014-007321 PMC4466605 26068509

[36] Beune EJAJ , Haafkens JA , Bindels PJE . Barriers and enablers in the implementation of a provider‐based intervention to stimulate culturally appropriate hypertension education. Patient Educ Couns. 2011;82(1):74‐80. 20303232 10.1016/j.pec.2010.02.015

[37] Davis AM , Kennedy D , Wong R , et al. Cross‐cultural adaptation and implementation of Good Life with osteoarthritis in Denmark (GLA:D™): Group education and exercise for hip and knee osteoarthritis is feasible in Canada. Osteoarthritis Cartilage. 2018;26(2):211‐219. 29146385 10.1016/j.joca.2017.11.005

[38] Bardosh KL , Murray M , Khaemba AM , Smillie K , Lester R . Operationalizing mHealth to improve patient care: A qualitative implementation science evaluation of the WelTel texting intervention in Canada and Kenya. Global Health. 2017;13(1):87. 29208026 10.1186/s12992-017-0311-z PMC5717811

[39] Denzin NK , Lincoln YS . Handbook of Qualitative Research. Thousand Oaks, CA: Sage Publications; 1984.

[40] Langley G , Nolan K , Nolan T , Norman C , Provost L . The Improvement Guide: A Practical Approach to Enhancing Organization Performance. San Francisco, CA: Jossey‐Bass; 2009.

[41] Lincoln Y , Guba E . Naturalistic Inquiry. Newbury Park, CA: Sage Publications; 1985.

[42] Guest G , MacQueen K , Namey E . Applied Thematic Analysis. Thousand Oaks, CA: Sage Publications; 2012.

[43] Saldana J . The Coding Manual for Qualitative Researchers, 3rd edn Thousand Oaks, CA: Sage Publications; 2015.

[44] Giacomini M , Cook DJ . Users' guides to the medical literature: XXIII. Qualitative research in health care: Are the results of the study valid? J Am Med Assoc. 2000;284(3):357‐362. 10.1001/jama.284.3.357 10891968

[45] Guba E , Lincoln Y . Fourth Generation Evaluation. Newbury Park, CA: Sage Publications; 1989.

[46] Department of Health and Human Services, Centers for Medicare and Medicaid Services . Medicare Program; Hospital Conditions of Participation: Requirements for Approval and ReApproval of Transplant Centers To Perform Organ Transplants. Baltimore, MD: Federal Register; 2007. 17450666

[47] United Network for Organ Sharing . Organ Procurement and Transplant Network Data. Richmond, VA: University Renal Research and Education Association; 2016.

[48] Artiga S , Foutz J , Damico A .Changes in health coverage by race and ethnicity since implementation of the ACA, 2013–2017; 2018 Retrieved from https://www.kff.org/disparities‐policy/issue‐brief/changes‐in‐health‐coverage‐by‐race‐and‐ethnicity‐since‐implementation‐of‐the‐aca‐2013‐2017

[49] Hall W , Chapman M , Lee K , et al. Implicit racial/ethnic bias among health care professionals and its influence on health care outcomes: A systematic review. Am J Public Health. 2015;105(12):e60‐e 76. 10.2105/AJPH.2015.302903 PMC4638275 26469668

[50] Kim JJ , Basu M , Plantinga L , et al. Awareness of racial disparities in kidney transplantation among health care providers in dialysis facilities. Clin J Am Soc Nephrol. 2018;13(5):772‐781. 29650714 10.2215/CJN.09920917 PMC5969478

[51] Johnson R . Using Data to Close the Achievement Gap: How to Measure Equity in Our Schools. Thousand Oaks, CA: Corwin Press; 2002.

[52] Bernal G , Adames C . Cultural adaptations: Conceptual, ethical, contextual, and methodological issues for working with ethnocultural and majority‐world populations. Prev Sci. 2017;18(6):681‐688. 28573426 10.1007/s11121-017-0806-0

[53] Finkler K . Physicians at Work, Patients in Pain: Biomedical Practice and Patient Response in Mexico. Oxford: Westview Press; 1991.

[54] Brennan Ramirez LK , Baker EA , Metzler M . Promoting Health Equity: A Resource to Help Communities Address Social Determinants of Health. Atlanta, GA: U.S. Department of Health and Human Services, Centers for Disease Control and Prevention; 2008 https://www.cdc.gov/nccdphp/dch/programs/healthycommunitiesprogram/tools/pdf/SDOH‐workbook.pdf Accessed June 10, 2020.

[55] Guerrero E , Fenwick K , Kong Y . Advancing theory development: Exploring the leadership‐climate relationship as a mechanism of the implementation of cultural competence. Implement Sci. 2017;12(1):133. 29137668 10.1186/s13012-017-0666-9 PMC5686798

[56] Internal Revenue Service . Individual Taxpayer Identification Number; 2019 https://www.irs.gov/individuals/individual‐taxpayer‐identification‐number. Accessed 10 May 2019.

[57] Campinha‐Bacote J . The Process of Cultural Competence in the Delivery of Healthcare Services: The Journey Continues (5th edn). Cinncinatti, OH: Transcultural C.A.R.E. Associates; 2007.

